# Salvage Therapy With Inotuzumab Ozogamicin in Relapsed/Refractory B‐ALL After CAR‐T Therapy and HSCT: A Case Series

**DOI:** 10.1002/cnr2.70601

**Published:** 2026-07-01

**Authors:** Honghu Li, Luxin Yang, Lizhen Liu, Xiaoyu Lai, Lixia Zhu, Kui Zhao, Qian Luo, He Huang, Yi Luo

**Affiliations:** ^1^ Bone Marrow Transplantation Center, the First Affiliated Hospital Zhejiang University School of Medicine Hangzhou China; ^2^ Liangzhu Laboratory Zhejiang University School of Medicine Hangzhou China; ^3^ Institute of Hematology, Zhejiang University Hangzhou China; ^4^ Zhejiang Province Engineering Research Center for Stem Cell and Immunity Therapy Hangzhou China; ^5^ Department of Nuclear Medicine, the First Affiliated Hospital, College of Medicine Zhejiang University Hangzhou China; ^6^ Department of Ultrasound in Medicine The Second Affiliated Hospital of Zhejiang University School of Medicine, Zhejiang University Hangzhou China

**Keywords:** allogeneic hematopoietic stem cell transplantation, B‐cell acute lymphoblastic leukemia, chimeric antigen receptor T‐cell therapy, immunotherapy, inotuzumab ozogamicin

## Abstract

**Background:**

Although inotuzumab ozogamicin (InO) has been approved as an effective agent for patients with refractory/relapsed (r/r) B‐cell acute lymphoblastic leukemia (B‐ALL), data in the context of post chimeric antigen receptor (CAR)‐T cell therapy and allogeneic hematopoietic stem cell transplantation (allo‐HSCT) relapse are lacking.

**Case:**

We report on a series of three heavily pretreated r/r B‐ALL patients who relapsed after CAR‐T therapy and allo‐HSCT and were salvage treated with InO. All achieved bone marrow (BM) remission with negative minimal residual disease (MRD) after the first InO cycle; of note, one patient with extensive extramedullary involvement before InO treatment experienced a remarkable tumor regression on PET/CT imaging. They all completed 2 cycles of InO. Two patients experienced relapse 8 and 9.5 months after InO initiation (one central nervous system relapse, the other breast relapse). Treatment‐related adverse events (AEs) were primarily hematologic and manageable. Crucially, no sinusoidal obstruction syndrome (SOS) developed in any of the patients during the whole course of InO therapy and beyond.

**Conclusion:**

InO appears to be a promising and well‐tolerated salvage regimen for inducing remission in heavily pretreated B‐ALL patients who have relapsed after both CAR‐T therapy and allo‐HSCT.

## 1 Introduction

The prognosis is discouraging for refractory/relapsed (r/r) B‐cell acute lymphoblastic leukemia (B‐ALL) characterized by low remission rate, short remission interval, high recurrence rate, and poor survival [1, 2] Conventional chemotherapy in adults with r/r B‐ALL yielded an overall complete remission (CR) rate of 31%–44% in first salvage of early relapses and only 18%–25% in salvage 2 or beyond [2, 3]. The emergence of immunotherapies, including bispecific antibody constructs, antibody conjugates and chimeric antigen receptor (CAR) T‐cell therapy, has revolutionized the treatment landscape. CAR‐T therapy in particular demonstrates impressive initial CR rates of 80%–90% [4]. Nevertheless, its long‐term efficacy is attenuated by a high relapse rate of 30%–60%, partly due to limited CAR‐T cell persistence [5, 6]. While consolidative allogeneic hematopoietic stem cell transplantation (allo‐HSCT) after CAR‐T therapy improves outcomes [7, 8], relapse remains the leading cause of post‐transplant mortality [9]. Outcomes for adult ALL patients who relapsed after HSCT were extremely poor, with a limited median overall survival (OS) of merely 5.5 months and estimated 1‐year, 2‐year and 5‐year OS rates around 30%, 16% and 8%, respectively [10, 11]. Not to say the prognosis of ALL relapse after both CAR‐T therapy and HSCT. Such population often resists to multiple drugs, in a state of severe immunodeficiency, infection and myelosuppression, leaving few therapeutic options. Novel agents to induce deeper remissions with less toxicity are urgently needed.

Inotuzumab ozogamicin (InO) is a CD22‐directed antibody‐drug conjugate (ADC) linked to the cytotoxic antibiotic calicheamicin [12, 13]. Once InO binds to CD22‐positive leukemic blasts, the ADC complex is promptly internalized and calicheamicin is released along with the ensuing cell apoptosis [12, 14]. Its “off‐the‐shelf” availability and T cell independent mechanism of action, particularly the absence of cytokine release syndrome (CRS) and immune effector cell‐associated neurotoxicity syndrome (ICANS), make it an appealing option [15]. Although the INO‐VATE trial has established its superiority over standard chemotherapy in r/r B‐ALL [16], its role in patients who relapse after both CAR‐T therapy and allo‐HSCT is not well defined. We present a case series of three patients treated with InO in this specific, highly challenging setting, for which we previously lacked evidence.

## 2 Case Series

Between September 2019 and October 2021, three consecutive patients with Philadelphia chromosome‐negative B‐ALL who relapsed after both CAR‐T therapy and allo‐HSCT were treated with InO monotherapy at our center. All three patients had confirmed CD22 expression on leukemic blasts, an ECOG performance status ≤ 3, and no active grade III‐IV GvHD. No other patients meeting these criteria were identified during this period. All three patients are reported in this case series.

### 2.1 Case 1

Case #1 is a 53‐year‐old female with newly diagnosed B‐ALL (03/2021). Leukemia fusion genotyping revealed an E2A::PBX1 and WT1 fusion (Table 1). After one course of VP (vincristine, prednisone)‐based chemotherapy, she achieved first CR (CR1) without.

**TABLE 1:** Patient characteristics and baseline data.

Case	Age	Sex	Blast in BM (%) at diagnosis	Chromosome at disease onset	Gene abnormities at diagnosis	WBC (×10^9^/L) in PB at diagnosis	CART target	Antigen expression post CAR‐T/HSCT repalse	Salvage therapies before HSCT	Disease status at HSCT
#1	53	F	87%	Normal	E2A::PBX1 WT1	43.9	CD19&CD22	CD19 + CD34‐CD10 + CD38 + CD22 + CD20‐CD58 + CD81‐CD45dim	Hyper CVAD +L‐Asp	MRD‐negative CR2
#2	16	F	77%	Complex	CEBPB::IGH KMT2D mutation NRAS mutation	10.1	CD19	CD19 + CD34 + CD10 + CD38dimCD22 + CD20dimCD58 + CD81‐CD45dim	None	MRD‐negative CR3
#3	29	F	73%	Normal	None	25.8	Dual CD20/CD22&CD19	CD19 + CD34 + CD10 + CD38 + CD22dimCD20dimCD58 + CD81 + CD45dim	Hyper CVAD+L‐asp	MRD‐negative CR4

Donor age (year) type, gender, HLA match	Conditioning regimen	Time from HSCT to neutrophil engraftment (days)	Time from HSCT to platelet engraftment (days)	Months from HSCT to relapse	HLA‐loss at post‐HSCT relapse	[blast% in BM] (CD22+) before InO	Extramedullary disease before InO
20, M, 5/10 son	MAC	17	19	6	HLA‐B13	20%	None
38, M, 5/10 father	MAC	14	15	< 5	None	80%	Multiple sites
33, F, 5/10 sister	MAC	15	15	16	HLA‐DQB1	83%	CNS

Abbreviations: BM, bone marrow; CAR‐T, chimeric antigen receptor T cell; CR, complete remission; F, female; HSCT, hematopoietic stem cell transplantation; Hyper CVAD, hyperfractionated cyclophosphamide, vincristine, doxorubicin, and dexamethasone; InO, Inotuzumab ozogamicin; L‐Asp, L‐asparaginase; MAC, myeloablative conditioning; MRD indicates minimal residual disease; PB, peripheral blood; WBC, white blood cell.

Detectable BM MRD (Figure 1). Then she was enrolled in the phase I, single center trial of sequential autologous CD19‐22 CAR‐T therapy. Two separate CAR‐T cell products (CD19 and CD22 CAR‐T cells) were sequentially infused to her within 3 months. However, BM relapse occurred 8 months after the first infusion of CD19 CAR‐T cell (01/2022). At relapse, BM smear displayed lymphoblasts accounted for 93%. Flow cytometry (FCM) indicated that the blast cells were positive for CD19, CD10, CD38, CD22, CD58, CD45dim. Afterwards, she received 2 cycles of Hyper CVAD (hyperfractionated cyclophosphamide, vincristine, doxorubicin, and dexamethasone) salvage chemotherapy, achieving MRD negative CR2 and bridging to allo‐HSCT. Conditioning for this allo‐HSCT was performed with cytarabine, busulfan and cyclophosphamide, and graft versus host disease (GvHD) prophylaxis was achieved with anti‐thymocyte globulin (ATG), methotrexate (MTX), mycophenolate mofetil (MMF) and cyclosporine. Nevertheless, 6 months after transplantation, she suffered a second BM relapse with the loss of HLA‐B13 (11/2022). She received 2 cycles of InO (cycle 1: total dose 1.8 mg/m2; cycle 2: total dose 1.5 mg/m2) and monthly intrathecal chemotherapy. After the first InO cycle, she achieved complete remission/complete remission with incomplete hematologic recovery (CR/CRi) status without detectable BM MRD. However, she experienced molecular progression (E2A::PBX1 from 0.033% to 8.52%) and later developed extramedullary relapse (EMR) with 37.34% of lymphoblasts in the cerebrospinal fluid (CSF) 8 months after the first cycle of InO (07/2023). Then she was subjected to repeated intrathecal chemotherapy until the achievement of MRD negativity in CSF and shifted to VICP (vincristine, idarubicin, cyclophosphamide and prednisone)‐based regimen.

**FIGURE 1:**
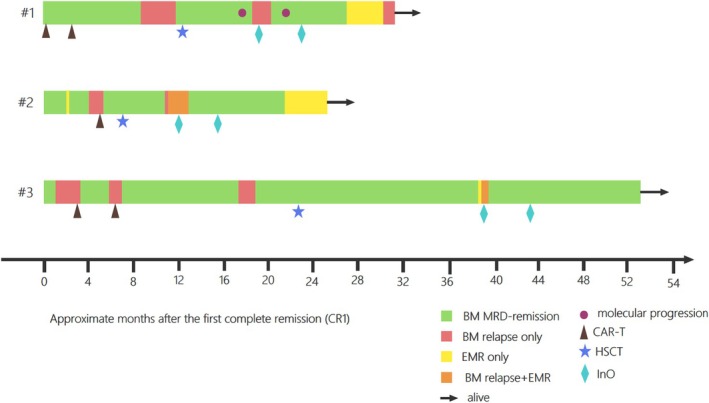
Swimmer plot of the three patients' clinical courses. The treatment timelines and responses for each patient before and after InO treatment are shown. Bars of different colors indicate time within specific disease conditions.

### 2.2 Case 2

Case #2 is a 16‐year‐old teenage girl with relapsed B‐ALL. Next‐generation sequencing revealed the presence of a CEBPB::IGH fusion, and gene mutations were positive for KMT2D and NRAS at diagnosis (11/2021) (Table 1). At first, she received 1 cycle of VICP induction chemotherapy and achieved MRD‐negative CR1 (Figure 1). Subsequently, she received another course of CAM (cyclophosphamide, mercaptopurine, cytarabine) as consolidation therapy but a month later experienced EMR with 40.472% of primitive B lymphocytes in the CSF while remaining BM remission status (02/2022). Therefore, she was subjected to repeated intrathecal chemotherapy and shifted to methotrexate and pegaspargase chemotherapy until the achievement of MRD negativity in CSF. Afterwards, she received a course of EA (etoposide, cytarabine) regimen but suffered a second disease recurrence manifesting as BM relapse (04/2022). Then the patient was enrolled in the phase I, single center trial of universal CD19‐targeted CAR‐T (CD19‐UCAR‐T) therapy. 3 weeks after treatment with CD19‐UCAR‐T cells, she reached CR status again and successfully bridged to allo‐HSCT after conditioning with cytarabine, busulfan and cyclophosphamide. GvHD prophylaxis was achieved with ATG, MTX, MMF and cyclosporine. However, a third recurrence (a combined multiple extramedullary and BM relapse) occurred within 5 months post‐transplant (12/2022). Positron emission tomography/computed tomography (PET/CT) scan indicated extensive extramedullary involvement (Figure 2A). BM smear showed lymphoblasts accounted for 80% and FCM suggested that the blast cells were positive for CD19, CD34, CD10, CD38dim, CD22, CD20dim, CD58, CD45dim. Because of recurrent relapse, she received 2 cycles of InO therapy (cycle 1: total dose 1.8 mg/m2; cycle 2: total dose 1.5 mg/m2). After the first InO cycle, she achieved BM remission without detectable BM MRD. Comparison of PET/CT scan before and after InO treatment indicated that most of the metabolically active lesions displayed a considerable decrease in size and fluorodeoxyglucose (FDG) uptake after one course of InO (Figure 2). However, 9.5 months after the first cycle of InO, she developed isolated EMR manifesting as breast infiltration identified via PET/CT examination and confirmed by biopsy analysis (09/2023). At the time of this relapse, her BM and CSF were found to be in remission status.

**FIGURE 2:**
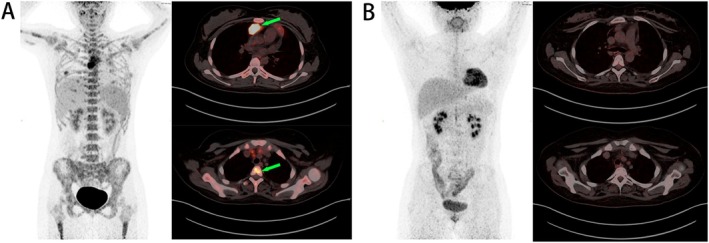
Positron emission tomography/computed tomography (PET/CT) Imaging for case 2. (A) Baseline PET/CT scan before InO treatment. The green arrows indicate hypermetabolic leukemic infiltration in the anterior mediastinum and extensive marrow cavity involvement. (B) A follow‐up PET/CT scan performed after the first cycle of InO treatment, demonstrating a remarkable resolution of the previously noted lesions, with significantly diminished size and FDG avidity, indicating a profound metabolic response. Abbreviation: † FDG, fluorodeoxyglucose.

### 2.3 Case 3

Case #3 is a 29‐year‐old female with relapsed B‐ALL. No specific fusion genes or gene mutations were found in her BM at diagnosis (09/2019) (Table 1). At first, she received 1 cycle of VICP induction chemotherapy and achieved MRD‐negative CR1 (Figure 1). Subsequently, she received another course of CAM as consolidation therapy but soon developed BM relapse (11/2019). A regimen of venetoclax combined with prednisone was given to her but showed nonresponse and nonremission. Therefore, she received bispecific CD20/CD22‐targeted CAR‐T therapy in the relapsed status. One month after infusion, she achieved CR2 but suffered an ultra‐early BM relapse 2 months later (04/2020). Afterwards, she was subjected to a second CD19‐UCAR‐T therapy and reached MRD‐negative CR3 2 weeks after infusion (05/2020). Her last CR3 status sustained more than 10 months before the occurrence of a third BM relapse (03/2021). BM smear displayed lymphoblasts accounting for 76%. FCM indicated that the blast cells were positive for CD19, CD34, CD10, CD38, CD22dim, CD20dim, CD58, CD81, and CD45dim. She then received 3 cycles of Hyper CVAD‐based salvage chemotherapy, achieving and remaining CR4 since the first cycle. Afterwards, she underwent allo‐HSCT (09/2021) after conditioning with cytarabine, busulfan, and cyclophosphamide, until a fourth BM relapse occurred 16 months post‐transplant (01/2023). HLA‐DQB1 loss was confirmed at post‐transplant relapse. Treatment with 2 cycles of InO was started (cycle 1: total dose 1.8 mg/m2; cycle 2: total dose 1.5 mg/m2). She achieved CR/CRi after the first cycle of InO and kept leukemia‐free until the latest follow‐up time (13.5 months). However, 1 month after the second cycle of InO, she suffered two epileptic seizures manifested by a sudden loss of consciousness, convulsions, eyes on the turn, and uroclepsia, which required pharmacological intervention.

In terms of safety issues, the treatment was well tolerated with a favorable toxicity profile. No patient died or discontinued medication from treatment‐emergent adverse events (AEs). It had a minor impact on liver function, with hypertransaminasemia and hyperbilirubinemia being of only grade 1/2 (G1/2) severity. Importantly, no patients developed GvHD or sinusoidal obstruction syndrome (SOS). The most common toxicity was pancytopenia. All 3 patients had ≥G3 leukocytopenia, neutropenia, and thrombocytopenia that requires white blood cell (WBC) booster and platelet transfusions. Two patients (case #2 and case #3) had ≥G3 anemia requiring transfusions of red blood cell (RBC). No evidence of damage in other systems and infection complication was detected. Details of toxic effects have been categorized, graded, and shown in Table 2.

**TABLE 2:** Adverse events suspected to be related to InO after treatment.

Patient	WBC	Neu	Hb	PLT	ALT	AST	TBil
#1	G3	G3	G2	G3	G1	G1	N
#2	G3	G4	G3	G4	G1	G1	N
#3	G4	G4	G3	G4	G1	G1	G2

Abbreviations: N, normal; ALT, alanine aminotransferase; AST, aspartate aminotransferase; Hb, hemoglobin; Neu, neutrophil; PLT, platelet; TBil, total bilirubin; WBC, white blood cell.

## 3 Discussion

We present three patients who relapsed with B‐ALL after having received prior CAR‐T therapy and allo‐HSCT. As shown in Figure 1, when treated with InO, the median line of salvage treatment was three, which indicated that these cases were refractory and high‐risk. Though heavily pretreated, all of them achieved BM remission with MRD negativity after 1 cycle of InO. Two patients (case #1 and case #2) relapsed again 8 and 9.5 months after InO initiation, respectively. The other patient was in persistent CR at the cut‐off time. Our series suggests that InO remains a highly effective and well‐tolerated salvage regimen in this challenging clinical scenario, capable of inducing deep remissions. Our findings are consistent with the known single‐agent activity of InO in r/r ALL, as seen in the INO‐VATE trial [16], but uniquely extend these observations to a specific post‐CAR‐T and allo‐HSCT failure context.

The management of relapse post‐transplant is evolving; allogeneic, donor‐derived CAR‐T therapies have shown promise. In a study from Zhang and colleagues, they demonstrated a CR rate of 79% in 43 B‐ALL patients relapsing post allotransplant after the use of donor‐derived CD19 CAR‐T cells, but CRS occurred in up to 38 subjects (88%) [17]. Of the patients who achieved CR without a secondary transplantation, the 5‐year OS and event free survival (EFS) rates reached 53.13% and 46.88%, respectively [18]. Our previous study also provided definitive evidence of benefits for donor‐derived CAR T cells (GC007g) in relapsed B‐ALL after allo‐HSCT [19], all 9 patients enrolled in the group achieved CR/CRi with negative MRD at 28 days after infusion. The 1‐year progression free survival (PFS) and OS were 77.8% and 85.7%, respectively. Another comparative study of donor‐derived CAR‐T therapy versus donor lymphocyte infusion (DLI) for relapsed B‐ALL after allo‐HSCT revealed that superior outcomes were observed in the CAR‐T group [20]. In our series, as two patients experienced HLA loss relapse, donor cell‐derived approaches—including DLI and donor CAR‐T cells—are likely to be ineffective, as the leukemic blasts have escaped allorecognition. In this context, non‐HLA‐restricted antibody‐based constructs like InO and blinatumomab [21], represent a mechanistically rational alternative.

In the randomized phase 3 trials, both single‐agent blinatumomab (TOWER trial) [22] and InO (INO‐VATE trial) [16] demonstrated significantly higher remission rates and improved survival compared with standard chemotherapy in r/r ALL, including in the subgroup of patients who had relapsed after prior allo‐HSCT. However, several considerations led us to favor InO over blinatumomab for our patients. First, the efficacy of blinatumomab is known to be diminished in patients with high tumor burden (defined as bone marrow blasts ≥ 50%) [23], and consistently associated with inferior outcomes in patients with pre‐treatment MRD > 1% in a real‐world study [24], whereas InO has shown consistent activity regardless of blast percentage [25]. Second, InO has demonstrated particular clinical activity in clearing extramedullary disease [25]. As our patients had either high marrow blast counts or extensive extramedullary disease, InO was preferred in this case series.

While all patients achieved MRD‐negative remission, two eventually relapsed, highlighting the limited durability of InO monotherapy. This observation echoes the INO‐VATE trial, where the median remission duration was only 4.6 months even among InO‐treated patients [16], underscoring the need for post‐InO consolidation or maintenance strategies. For patients relapsing after allo‐HSCT without HLA loss, the combination of InO and DLI has shown promise in a small retrospective series, with a median RFS of 12.0 months [26], a prospective trial of this approach (NCT06861348) is also ongoing at our center. However, for patients with confirmed HLA loss, donor‐derived cellular therapies including DLI are mechanistically ineffective, and a second allo‐HSCT from a different mismatched donor may represent the only curative alternative [27]. For patients ineligible for a second transplant, combining InO with low‐intensity chemotherapy and/or blinatumomab is another logical approach. This strategy has yielded improved outcomes compared with InO monotherapy in broader r/r ALL populations [28, 29], with the most recent dose‐fractionated InO plus sequential blinatumomab regimen achieving a 3‐year OS rate of 40% and a median OS of 17 months [30]. Notably, a subset of patients in these studies had undergone prior allo‐HSCT, supporting the feasibility of this approach in the post‐transplant setting.

With respect to the safety profile, hematopoietic toxicity and hepatotoxicity are two notable concerns in InO therapy. However, SOS, one of the most serious adverse effects previously reported [31], did not happen in our study, which may attribute to patient‐specific factors, the prophylactic use of ursodeoxycholic acid, and the limited number of treatment cycles. The most common toxicity observed was pancytopenia, though significant, was expected given the pre‐existing at least monolineage cytopenia in all patients before InO administration, which makes ascribing hematologic toxicity solely to InO difficult. Liver toxicities were mild and effectively managed with standard hepatoprotective therapy. No evidence of GvHD and fatal infection events were observed relating to InO, suggesting a well‐tolerant feature of InO.

The key strength of this report is its focus on a well‐defined, extremely high‐risk patient population for which prospective data are virtually non‐existent. The principal limitations are inherent to the case series design: the small sample size (*n* = 3) and retrospective nature preclude broad generalizations and meaningful statistical survival analysis. Despite these limitations, this real‐world experience addresses a critical clinical gap and may inform treatment decisions for similarly situated patients until larger, prospective studies become available.

## 4 Conclusion

In summary, our small case series provides preliminary evidence of efficacy and an acceptable safety profile of InO in patients with r/r B‐ALL who particularly relapsed after both CAR‐T therapy and HSCT, rendering InO to be an encouraging salvage regimen in this challenging scenario. It also holds promise as a bridging therapy to subsequent treatment, like a second transplant. Larger, multi‐center prospective studies are required to confirm these findings and define the optimal role of InO‐based combination strategies in this difficult context.

## Author Contributions

**Honghu Li:** conceptualization, writing – review and editing, writing – original draft, visualization. **Lixia Zhu:** conceptualization. **Xiaoyu Lai:** conceptualization. **Luxin Yang:** investigation, formal analysis, methodology, data curation. **He Huang:** writing – review and editing, validation. **Kui Zhao:** project administration, resources. **Lizhen Liu:** conceptualization. **Yi Luo:** writing – review and editing, validation. **Qian Luo:** supervision.

## Funding

This work was supported by the grants from the National Natural Science Foundation of China (82200233, 82400253), Zhejiang Provincial Natural Science Foundation of China (LTGY24H080002, LQ24H080001).

## Ethics Statement

This study was approved by the Institutional Review Board (IRB) of the First Affiliated Hospital of Zhejiang University School of Medicine (IIT20250318B‐R1).

## Consent

Written informed consent was obtained from patients themselves or parents of patients under 18 years of age for the publication of this case series, including all clinical details and images.

## Conflicts of Interest

The authors declare no conflicts of interest.

## Data Availability

The data that support the findings of this study are available from the corresponding author upon reasonable request.
